# Bilateral transient visual loss and meningeal irritation signs following retrobulbar anesthesia

**DOI:** 10.3205/oc000065

**Published:** 2017-06-22

**Authors:** Melih Ustaoglu, Nilgun Solmaz, Feyza Onder

**Affiliations:** 1 Sisli Hamidiye Etfal Training and Research Hospital, Ophthalmology Clinic, Istanbul, Turkey; 2 Haseki Training and Research Hospital, Ophthalmology Clinic, Istanbul, Turkey

**Keywords:** bilateral transient visual loss, meningeal irritation signs, optic nerve sheath penetration, retrobulbar anesthesia

## Abstract

A 62-year-old man underwent an uneventful cataract surgery in the left eye following retrobulbar anesthesia. Fifteen minutes after the surgery, the patient had visual loss in his right (unoperated) eye, headache, dizziness, nausea, and vomiting. The bandage on the left (operated) eye was removed and the initial ophthalmic examination revealed bilateral dilated pupils with absence of light perception. His fundus examination and vital signs were unremarkable. Immediately, a computerized tomography (CT) was performed to scan both orbit and brain. The orbit CT revealed air bubbles within the left optic nerve sheath, which confirmed inadvertent injection and administration of anesthetic medications into the optic nerve sheath. Within three hours, meningeal irritation signs recovered spontaneously and visual acuity improved to 20/20 in the right eye and 20/40 in the left eye.

## Introduction

In modern cataract surgery, the most popular anesthesia technique is topical anesthesia. In addition to this, retrobulbar anesthesia is still preferred for anxious individuals and uncooperative patients if akinesia is required. Although retrobulbar anesthesia provides a better surgical comfort and stability during surgery, it has various complications including chemosis, conjunctival hemorrhage, vessel damage leading to retrobulbar hemorrhage, extra-ocular muscle damage, globe perforation and optic nerve damage [1].

Here, we report a case presented with bilateral transient visual loss and meningeal irritation signs (headache, nausea, vomiting, and dizziness) due to optic nerve sheath penetration and the subsequent reflux of the anesthetic medications into the optic chiasm following retrobulbar anesthesia.

## Case description

A 62-year-old man presented with low vision in his left eye. On ophthalmic examination, the best corrected visual acuity (BCVA) was 20/20 for the right eye and 20/60 for the left eye. Anterior segment examination revealed grade III nuclear cataract in the left eye, fundus examination and intraocular pressure were unremarkable. The axial length of the left eye was 22.80 mm and there was no staphyloma. 

After the diagnosis of senile nuclear cataract in the left eye, the patient was prepared for phacoemulsification and intraocular lens implantation surgery under retrobulbar anesthesia. Retrobulbar anesthesia was preferred because the patient seemed anxious and incompatible. The anesthetic medication, 4 ml combination of lidocaine 1% and bupivacaine 0.25% without hyaluronidase, was administered by a first-year ophthalmology resident with a 23 gauge, 31 mm long needle through the infratemporal quadrant, while the patient was in supine position and looking straight ahead. Then additional 2 ml of the anesthetic mixture was injected with the same method five minutes after the first injection, since sufficient akinesia could not be obtained.

The cataract surgery was completed uneventfully in fifteen minutes, and then the patient was transferred to his room. On the follow-up visit fifteen minutes after the surgery, the patient had visual loss in his right (unoperated) eye, dizziness, nausea, and vomiting. The bandage on the left (operated) eye was removed and the initial ophthalmologic examination revealed bilateral dilated pupils with absence of light perception. The fundus examination was unremarkable bilaterally, which ruled out arterial occlusion and any retinal pathology. Systemic evaluation revealed a blood glucose level of 164 mg/dL, a body temperature of 37.1 °C, an arterial blood pressure of 110/ 80 mmHg, and a heart rate of 64 beats per minute. There was no abnormality in electrocardiogram.

**Figure 1:**
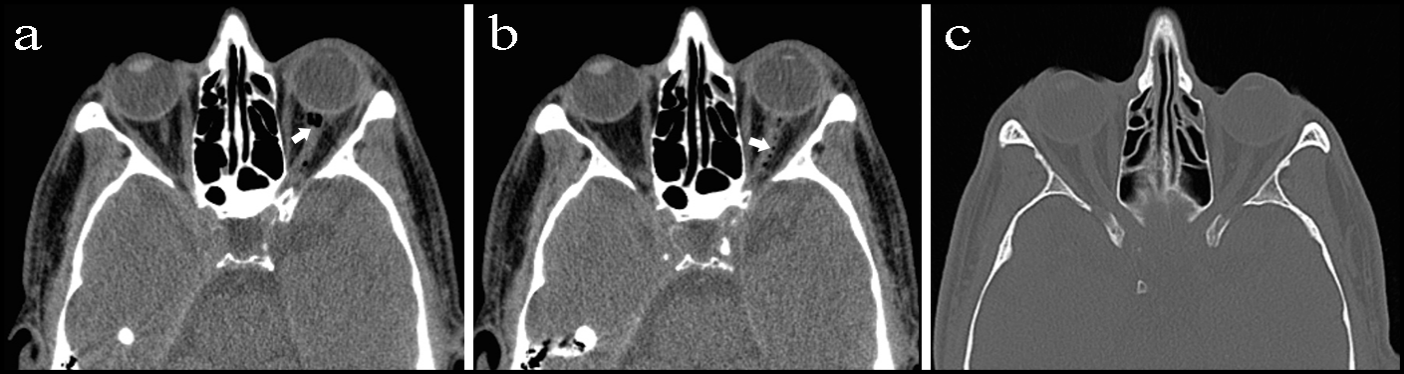
Computerized tomography (CT) images of the optic nerve sheath penetration (a) Air bubbles within the left (operated) optic nerve indicate optic nerve sheath penetration (arrow). (b) Movements of air bubbles to the chiasm (arrow). (c) The follow-up CT scan at day 3 shows disappeared air bubbles in the left optic nerve sheath.

After the initial ophthalmoscopic and systemic evaluation, a computerized tomography (CT) was performed to scan both orbit and brain and it showed round hypointense images corresponding to air bubbles within the optic nerve of the left eye (Figure 1a,b (Fig. 1)). Based on these air images within the optic nerve and existing symptoms, the patient was diagnosed with optic nerve sheath penetration following retrobulbar anesthesia. The patient started to recover within an hour and visual loss as well as meningeal irritation signs regressed gradually. After three hours following the administration of retrobulbar anesthesia, the BCVA of the patient improved to 20/20 in the right eye and 20/40 in the left eye. At the follow-up visit three days after the surgery, the BCVA of the patient was 20/20 bilaterally, and the orbital CT scan showed disappeared air images within the optic nerve sheath (Figure 1c (Fig. 1)).

## Discussion

Retrobulbar anesthesia is an ophthalmic regional anesthesia that provides sufficient analgesia and akinesia. However, since the needle is introduced into the intraconal space, retrobulbar anesthesia has serious complications such as extraocular muscle damage, retrobulbar hemorrhage, globe perforation, and optic nerve sheath penetration [1].

Optic nerve sheath penetration following retrobulbar anesthesia is a rare and severe complication. Injection of anesthetic medications into the subdural space can lead to bilateral transient visual loss and severe neurologic complications. The most accepted theory of bilateral transient visual loss is the inadvertent injection to the optic nerve sheath and reflux of anesthetic medications to the chiasm. The bilateral visual loss following optic nerve sheath penetration improves in accordance with the half-life of the anesthetic medications. In the literature, the authors reported that the visual acuity of the patients recovered approximately in ninety minutes to three hours subject to composition and quantity of the anesthetic medications [2], [3], [4]. The visual loss of our patient also recovered totally in three hours. Furthermore, there is only one report in the literature in which air bubbles within the optic nerve were shown by orbital CT [4]. To the best of our knowledge, our patient is the second case in which air bubbles within the optic nerve were demonstrated by orbital CT.

Central nervous system symptoms following optic nerve sheath penetration range from mild meningeal irritation signs to respiratory arrest and coma. Nicoll et al. [5] reported that sixteen out of six thousand consecutive patients (1 in 375) in whom retrobulbar anesthesia was performed developed neurologic symptoms such as drowsiness, blindness of contralateral eye, vomiting, respiratory depression, apnea, convulsion, unconsciousness, and cardiopulmonary arrest. The time up to the onset of symptoms ranged from 2 to 40 minutes (average 8 minutes). They also reported that hypotension and bradycardia were the first changes of the circulatory system. Therefore, all patients must be monitored during the surgery to provide early detection of neurologic complications. In our patient, we observed only mild meningeal irritation signs (headache, dizziness, nausea, and vomiting) half an hour after administration of the retrobulbar anesthesia and these signs recovered within three hours as well as the visual loss. We also did not observe any change in blood pressure and heart rate during the surgery. We believe that optic nerve sheath penetration occurred following the second injection in which only 2 ml of anesthetic medications was given. Therefore, injection of less anesthetic medication into the optic nerve sheath may explain why mild neurologic symptoms were observed in our patient.

This was the first case of optic nerve sheath penetration following retrobulbar anesthesia in our clinic, in which retrobulbar anesthesia is still preferred if patients’ akinesia is required. We estimate that the first injection we gave might have changed the position of the optic nerve in the orbital space and facilitated the optic nerve sheath penetration in our patient. 

## Conclusion

Optic nerve sheath penetration is a very severe complication of retrobulbar anethesia. In this condition, the patients must be informed that bilateral visual loss and neurologic symptoms are transient in accordance with half-lives of anesthetic medications and they must be observed intensively until all the symptoms recover.

## Notes

### Competing interests

The authors declare that they have no competing interests.

### Patient consent

The patient consented to the submission of this manuscript.
